# Development and Proof-of-Concept Analytical Validation of a HotStart Loop-Mediated Isothermal Amplification (LAMP) Assay Targeting the UL1 Gene for *Macacine alphaherpesvirus* 1 (McHV-1)

**DOI:** 10.3390/vetsci13090951

**Published:** 2026-09-12

**Authors:** Yang Xiao, Lianxiang Guo, Jingyi Zhang, Zhang Zhang, Ziwen Long, Hu Liu, Yaoming Li, Rong Bao

**Affiliations:** 1 Changde Vocational Technical College, Changde 415000, China; 2 Suzhou Xishan Biotech Inc., Suzhou 215021, China; 3 Center for Animal Experiment/ABSL-3 Laboratory, Wuhan University, Wuhan 430071, China; 4 Applied Biotechnology Research Center, Wuhan University of Bioengineering, Wuhan 430415, China

**Keywords:** loop-mediated isothermal amplification, analytical validation, visual detection, *Macacine alphaherpesvirus* 1, UL1 gene, B virus, *Macaca mulatta*

## Abstract

*Macacine alphaherpesvirus* 1 (McHV-1, commonly known as Monkey B Virus) is a fatal zoonotic pathogen naturally prevalent in macaques. In this study, we developed and conducted a proof-of-concept analytical validation of a HotStart Loop-Mediated Isothermal Amplification (LAMP) assay targeting a conserved, moderately GC-rich (61–62%) region of the UL1 gene in McHV-1. The reaction was optimized at 70 °C using engineered HotStart Bst 3.2/4.2 DNA Polymerase, tracked via real-time fluorescence and closed-tube visual colorimetric detection (pre-added L-HNB dye) without post-amplification tube opening to reduce the risk of post-amplification aerosol carry-over contamination. The assay achieved a preliminary practical analytical limit of detection (analytical LOD) of 100 copies/reaction (3/3 replicates) within a 15–30 min diagnostic cutoff window, matching the practical sensitivity of reference qPCR. Strict analytical specificity was demonstrated against a comprehensive panel of human herpesviruses (HHV-1 through HHV-5) and host macaque genomic DNA. As a single-target analytical prototype, this visual HotStart LAMP platform provides a rapid, equipment-free molecular tool with promising potential for McHV-1 surveillance in resource-limited settings and macaque breeding facilities upon further clinical validation.

## Simple Summary

Monkey B Virus is a zoonotic pathogen that naturally exists in macaque monkeys and poses a potential risk of fatal infection to humans upon transmission through bites or scratches. Currently, B virus detection requires expensive machines and well-trained technicians in a laboratory. Our method uses a constant warm temperature and a special chemical reaction that produces a clear color change that can be seen with the naked eye—from deep violet to bright yellow-green—when the virus is present. In our experiments using purified target DNA and spiked saliva specimens, this new test detected the viral target within 15 min and was as accurate as the much slower and more complex lab methods. Because this test is quick, easy to use, and does not rely on expensive instruments, it holds promise for future deployment in monkey breeding facilities and resource-limited regions. However, because this study was conducted using laboratory-prepared samples rather than naturally infected specimens, additional testing on real clinical samples will be needed before this method can be used for infection diagnosis.

## 1. Introduction

McHV-1, previously referred to as B virus (BV), is an enzootic *alphaherpesvirus* whose natural hosts comprise all 17 species of macaques (*Macaca* spp.) [1,2]. While infections in these natural hosts are typically asymptomatic, zoonotic transmission to humans causes fatal encephalomyelitis, with a mortality rate of approximately 70–80% in untreated cases [3,4,5]. Although cynomolgus macaques (*Macaca fascicularis*) are widely used in biomedical research, lethal McHV-1 strains are predominantly associated with rhesus macaques—which harbor the greatest diversity of McHV-1 strains [6]—as well as closely related species such as Japanese macaques [7].

Although historically considered a rare occupational hazard, the recent emergence of sporadic human infections has elicited significant global concern. Chronologically, the first human case was reported in Japan in 2019 [8], followed by a fatal transmission in Beijing, China, in 2021 [9,10]. Most recently, in 2024, a critical case was identified in Hong Kong following an attack by wild macaques [4,11,12].

These alarming incidents underscore the urgent need for rapid, field-deployable diagnostic tools to mitigate exposure risks among animal care personnel, laboratory workers, and vulnerable populations.

Currently, qPCR remains the gold standard for McHV-1 detection. However, it relies on expensive thermal cyclers, lengthy multi-step temperature protocols, and highly trained personnel, severely limiting its application in resource-limited or field environments such as breeding farms. Loop-mediated Isothermal Amplification (LAMP) serves as a rapid and simplified alternative. Nevertheless, a major challenge in detecting McHV-1 is its exceptionally high genomic GC content (~75%) [13], which heavily complicates isothermal amplification and increases the propensity for primer dimer formation.

To overcome this bottleneck, we designed a HotStart LAMP assay targeting a lower-GC (61–62%, nt 5321–5640) region of the UL1 gene [7], which serves as an analytical proof-of-concept prototype for robust McHV-1 DNA detection.

## 2. Materials and Methods

### 2.1. Ethics Statement

Macaque saliva matrices, utilized as background material for mock sample preparation, were collected from a breeding facility in Yibin, Sichuan. All animal procedures and sample collection protocols were conducted in strict accordance with animal welfare guidelines and were approved by the Institutional Animal Care and Use Committee (IACUC) of the Center for Animal Experiment/ABSL-3 Laboratory, Wuhan University (Protocol Review No. 20260435).

### 2.2. Phylogenetic Analysis and Primer Design

**Figure 1:**
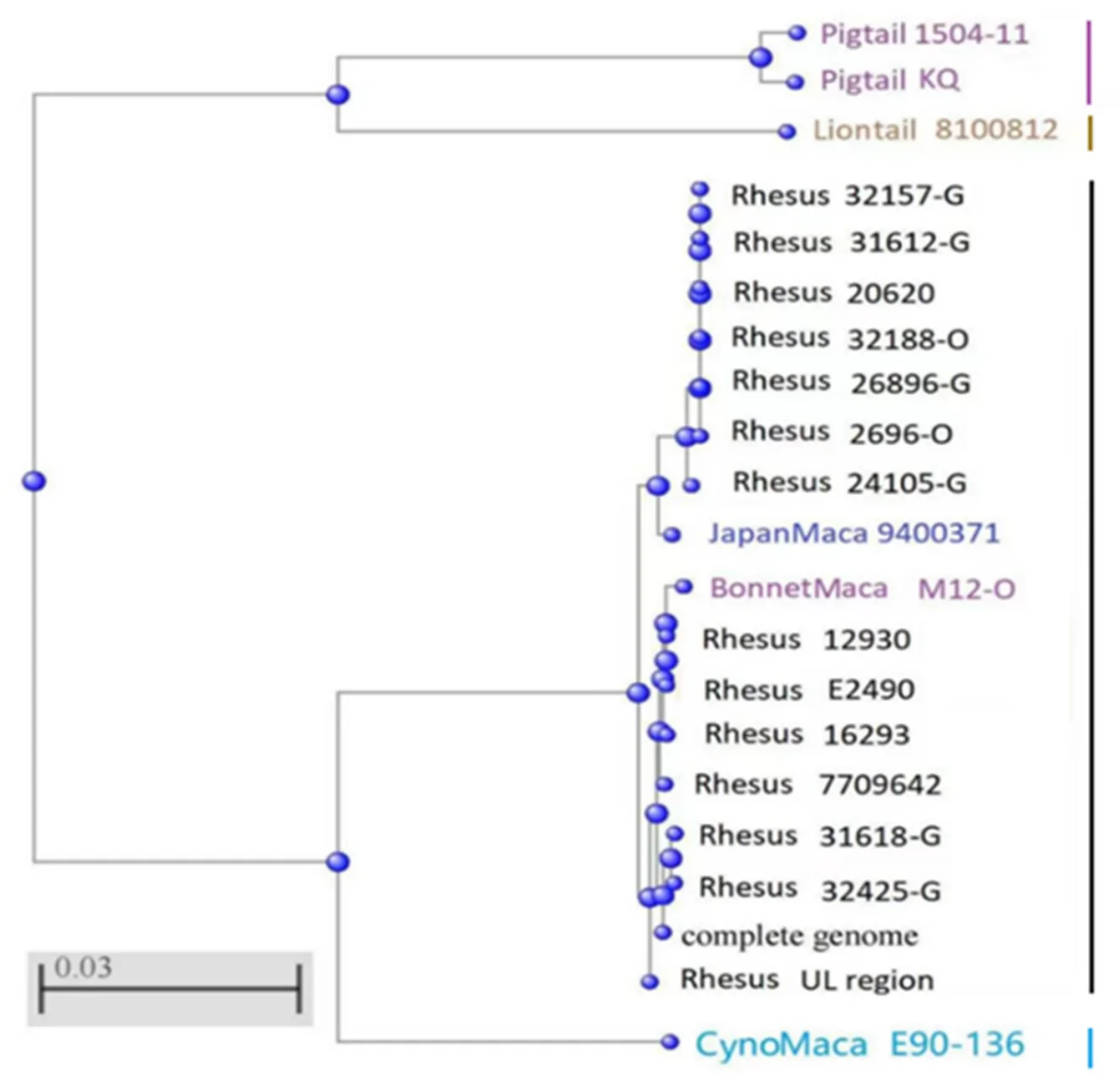
Phylogenetic analysis of the McHV-1 *UL1* gene. The phylogenetic tree, constructed using reference sequences retrieved from the NCBI Virus database, illustrates the evolutionary relationships between the standard E2490 strain and various macaque-derived isolates. The distinct cluster (highlighted in the box) underscores the high genetic conservation of the *UL1* gene across multiple macaque species (including *M. mulatta*, *M. fascata* and *M. radiata*).

**Figure 2:**
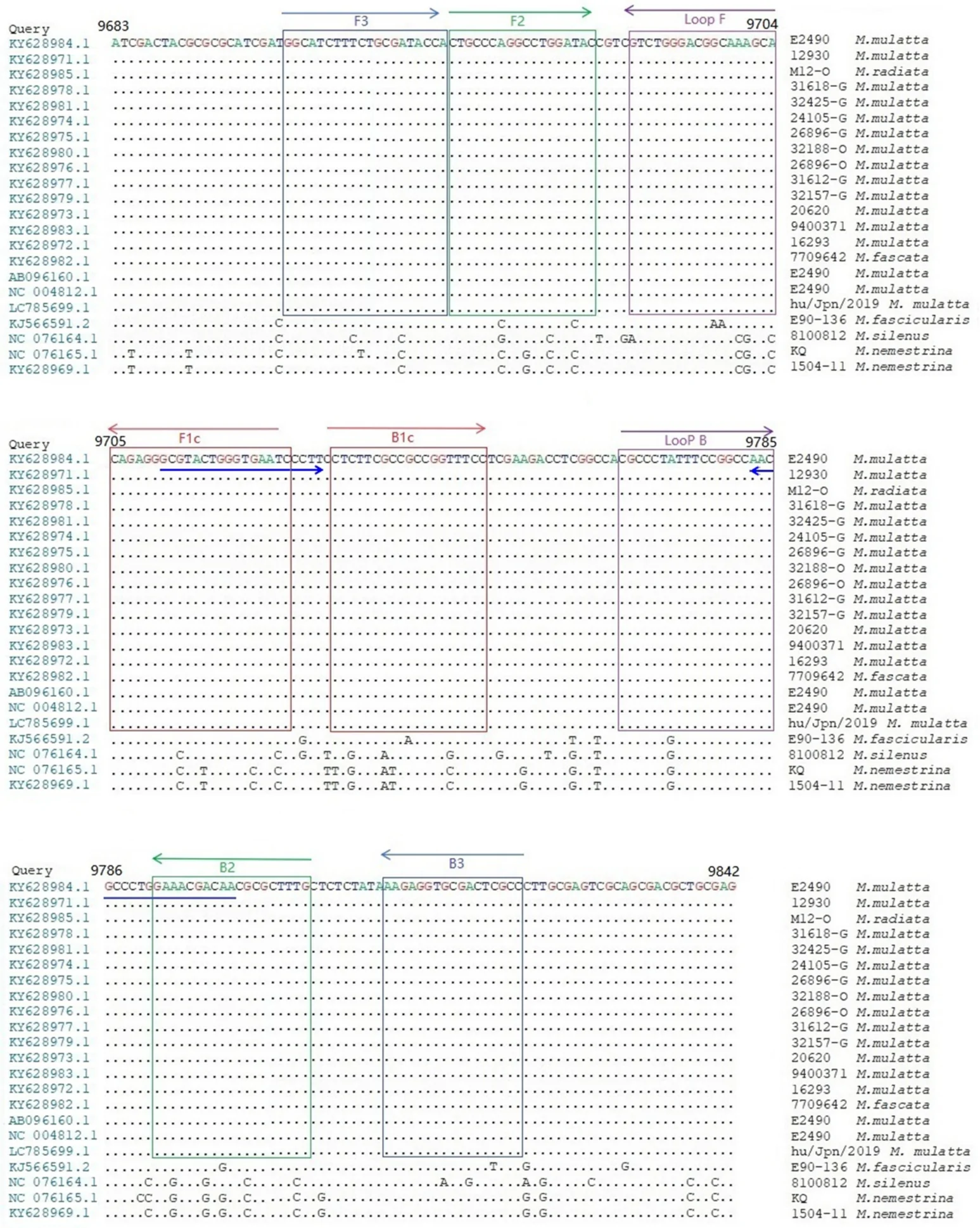
Schematic representation of the LAMP and qPCR primer sets alongside genomic sequence alignment. The upper panel delineates the binding sites and relative orientations of the six McHV-1 *UL1*-specific LAMP primers (F3, B3, FIP, BIP, LF, and LB; sequences provided in Table 1). The lower panel illustrates the co-localization of the qPCR primer pair (*UL1*-242), where the forward primer (91F) overlaps with the F1c region and the reverse primer (91R) maps to its respective complementary locus. Sequence alignment across diverse primate herpesvirus strains confirms high genetic conservation within the target region among major McHV-1 isolates (predominantly *Rhesus macaque*). Notably, strategic mismatches identified in non-target species and specific macaque strains (e.g., *M. fascicularis*, *M. silenus*, and *M. nemestrina*) are highlighted, ensuring the high analytical specificity of the developed assay.

The extracted genomic DNA of McHV-1 (strain E2490) used for assay validation was provided by Suzhou Xishan Biotech Inc. The complete genome of the McHV-1 E2490 strain (GenBank accession no. KY628984.1) served as the reference sequence. Prior to primer design, phylogenetic analysis was conducted to elucidate the evolutionary relationships between McHV-1 and other macaque herpesvirus strains (Figure 1). Guided by these genomic insights, LAMP primers targeting highly conserved domains within the *UL1* gene were specifically designed using PrimerExplorer V5 software to ensure robust analytical specificity (Figure 2). 

### 2.3. Quantitative PCR (qPCR) Assay 

To establish a reference for sensitivity and viral load quantification, a SYBR Green-based qPCR assay was performed using the Hieff^®^ qPCR SYBR Green Master Mix (Low Rox Plus) (Yeasen, Shanghai, China). The primers *UL1*-242 91F (5′-GCGTACTGGGTGAATCCCTT-3′) and 91R (5′-TTGTCGTTTCCAGGGCGTT-3′) were utilized to amplify a specific segment of the *UL1* gene (Figure 2). The 20 μL reaction mixture comprised 10 μL of 2× Master Mix, 0.4 μL of each primer (yielding a 0.2 μM final concentration), and 1 μL of the DNA template. Thermal cycling was conducted on a QuantStudio 3 system with the following protocol: initial denaturation at 95 °C for 5 min, followed by 40 cycles of 95 °C for 10 s and 60 °C for 34 s. A standard curve was generated using 10-fold serial dilutions of the pUC57-*UL1* plasmid to accurately quantify the viral DNA copy numbers.

**Table 1:** Sequences of the LAMP primers targeting the McHV-1 *UL1* gene (192 bp).

Primers	Sequence
F3	GGCATCTTTCTGCGATACCA
B3	GGCGAGTCGCACCTCTT
FIP	GATTCACCCAGTACGCCCTCTG CTGCCCAGGCCTGGATAC
BIP	CTCTTCGCCGCCGGTTTCC CAAAGCGCGTTGTCGTTTC
LF	TGCTTTGCCGTCCCAGAC
LB	CGCCCTATTTCCGGCCAAC

### 2.4. HotStart LAMP Reaction and Monitoring 

The 70 °C HotStart LAMP assay was established and evaluated in two distinct reaction formulations to address different diagnostic needs:

(1) Real-time fluorescence formulation (25 μL total volume): The mixture contained 1× HotStart Isothermal Reaction Buffer supplemented with an additional 6 mM Mg^2+^, 1 mM of each dNTP, 1.6 μM each of internal primers FIP and BIP, 0.2 μM each of outer primers F3 and B3, 0.4 μM each of loop primers LF and LB, 1× LAMP Fluorescent Dye (B1700, New England Biolabs, Ipswich, MA, USA), 8 U-engineered aptamer-based HotStart Bst 3.2 DNA Polymerase (HaiGene, Harbin, China), and 2 μL of DNA template. Real-time fluorescence amplification was monitored at 70 °C for 60–70 min on a QuantStudio 3 system (Thermo Fisher Scientific, Waltham, MA, USA) or a portable isothermal platform (YP-R16). The fluorescence threshold was set at 10 standard deviations above the baseline fluorescence of initial cycles (cycles 3–8), with a strict 30 min diagnostic cutoff time.
(2) Visual L-HNB colorimetric formulation (25 μL total volume): The reaction comprised identical concentrations of reaction buffer, Mg^2+^ (6 mM final), dNTPs, primers, and HotStart Bst 4.2 DNA polymerase as above but omitted the fluorescent dye and pre-incorporated 120 μM Leuco-Hydroxynaphthol Blue (L-HNB) indicator dye prior to amplification. Reactions were performed in sealed, closed-tube format and incubated at 70 °C in a standard water bath or heating block for 30 min, without post-amplification tube opening to reduce the risk of post-amplification aerosol carry-over contamination. Color transition from deep violet (negative) to yellow-green (positive) was directly inspected by the naked eye.

To minimize contamination risks, rigorous physical workflow separation was maintained between reagent preparation, template addition, and amplification/detection areas, coupled with aerosol-barrier filter tips and regular UV irradiation.

### 2.5. Sensitivity and Specificity

To determine and compare the analytical sensitivity, or LOD, of the developed HotStart LAMP assay and the reference qPCR, 10-fold serial dilutions of both the recombinant pUC57-UL1 plasmid and extracted McHV-1 genomic DNA were prepared (encompassing a concentration range from 106 to 101 copies/μL). Each dilution iteration was evaluated in triplicate (*n* = 3) across both platforms within a predefined diagnostic cutoff window. The analytical LOD was defined as a preliminary practical analytical LOD representing the lowest dilution level yielding 100% positive detection (3/3 replicates) within the defined cutoff time, rather than a statistically modeled 95% Probit LOD. Furthermore, to thoroughly assess analytical specificity, a cross-reactivity panel was assembled comprising host macaque genomic DNA and a spectrum of non-target viral pathogens. All viral nucleic acids utilized in this evaluation were extracted using the Viral Genomic DNA/RNA Extraction Kit (Nanjing Jiancheng Bioengineering Institute, Nanjing, China). To ensure taxonomic rigor regarding the non-target human herpesviruses (HHVs), commercially available Liquid Internal Quality Control for HHV DNA (Types 2–5) was employed alongside standard HHV-1 isolates. The specificity panel—comprising HHV-1, HHV-2, HHV-3, HHV-4, and HHV-5—was subsequently tested in parallel using both the LAMP and qPCR assays to benchmark the diagnostic fidelity of the novel assay against the gold standard.

### 2.6. Validation Using Spiked Mock Samples

To evaluate the analytical matrix tolerance of the assay prototype in complex biological fluids, mock spiked specimens were prepared using unpurified macaque saliva as the background matrix. Saliva samples were collected from healthy, McHV-1-seronegative rhesus macaques housed at a breeding facility in Yibin, Sichuan, China. To purposefully stimulate salivary secretion while facilitating safe handling, the macaques were administered an intravenous (IV) injection of ketamine (10 mg/kg). Saliva was subsequently harvested using Salivette^®^ collection tubes (Sarstedt, Nümbrecht, Germany) and centrifuged at 1000 *g* for 2 min to recover the liquid matrix. To evaluate matrix tolerance against endogenous inhibitors, aliquots of crude saliva were spiked with McHV-1 genomic DNA (kindly provided by Lianxiang Guo, Suzhou Xishan Biotech Inc., Suzhou, China). For each macaque saliva specimen (*n* = 7), testing was conducted in parallel with a matched unspiked matrix control. These mock samples were evaluated in triplicate to assess whether crude biological matrix components exerted inhibitory interference on the HotStart LAMP kinetics or the L-HNB visual color transition.

## 3. Results

### 3.1. Assay Sensitivity (LOD)

**Figure 3:**
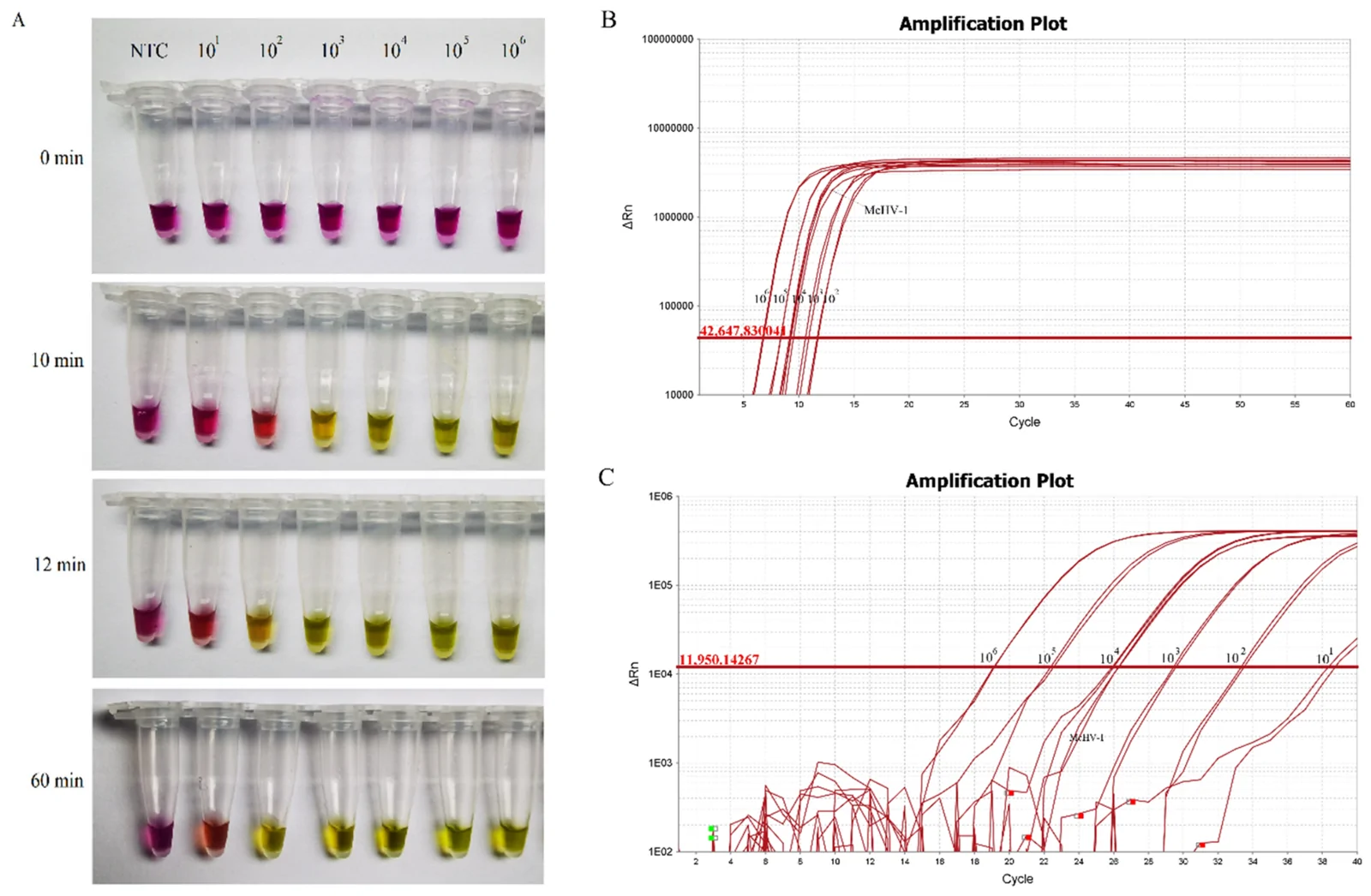
Analytical sensitivity and rapid amplification kinetics of the HotStart LAMP assay. The LOD was evaluated using 10-fold serial dilutions of the target plasmid ranging from 10^6^ down to 10^1^ copies/reaction. (**A**) Visual detection of the LAMP products using L-HNB dye at 0, 10, 12 and 60 min. The no-template control (NTC) remained violet, whereas reactions containing target templates gradually transitioned to yellow-green. At 60 min, the assay successfully distinguished down to 10 copies/reaction. (**B**) Corresponding real-time fluorescence amplification plot generated on a real-time PCR instrument using the B1700 fluorescent dye (NEB #B1700) incubated at 70 °C. Note: The x-axis represents the reaction time in minutes (recorded as “Cycles” by the instrument software, where the programmed two-step protocol of 30 s per step mathematically equates 1 cycle to 1 min). The plot enables precise quantification of the reaction kinetics, highlighting a remarkably short time-to-positivity (~10 min) for the target detection. (**C**) Standard curve of the reference qPCR assay establishing the quantitative baseline. The linear regression curve was generated by plotting the cycle threshold (Ct) values against the log_10_-transformed plasmid copy numbers (10^6^ to 10^1^ copies/reaction).

To evaluate the comparative analytical sensitivity of the developed HotStart LAMP assay, 10-fold serial dilutions of the recombinant plasmid template (ranging from 10^6^ to 10^1^ copies/reaction) were evaluated in triplicate in parallel with the reference qPCR. Under this head-to-head comparison, the HotStart LAMP assay demonstrated a preliminary practical analytical LOD of 100 copies/reaction, achieving 100% detection (3/3 replicates) within a standard 15 min diagnostic window, matching the practical sensitivity of the reference qPCR methodology (Figure 3C). Real-time fluorescence tracking (Figure 3B) confirmed rapid amplification kinetics under 70 °C isothermal conditions, where exponential signal generation for high-copy templates initiated within approximately 10 min. Endpoint visual detection using L-HNB (Figure 3A) similarly confirmed positive yellow-green transition at 100 copies/reaction within 15–20 min. While prolonged incubation (up to 60 min) allowed for detection down to 10 copies/reaction in both formats, 100 copies/reaction was defined as the preliminary practical analytical LOD.

### 3.2. Specificity and Cross-Reactivity

**Figure 4:**
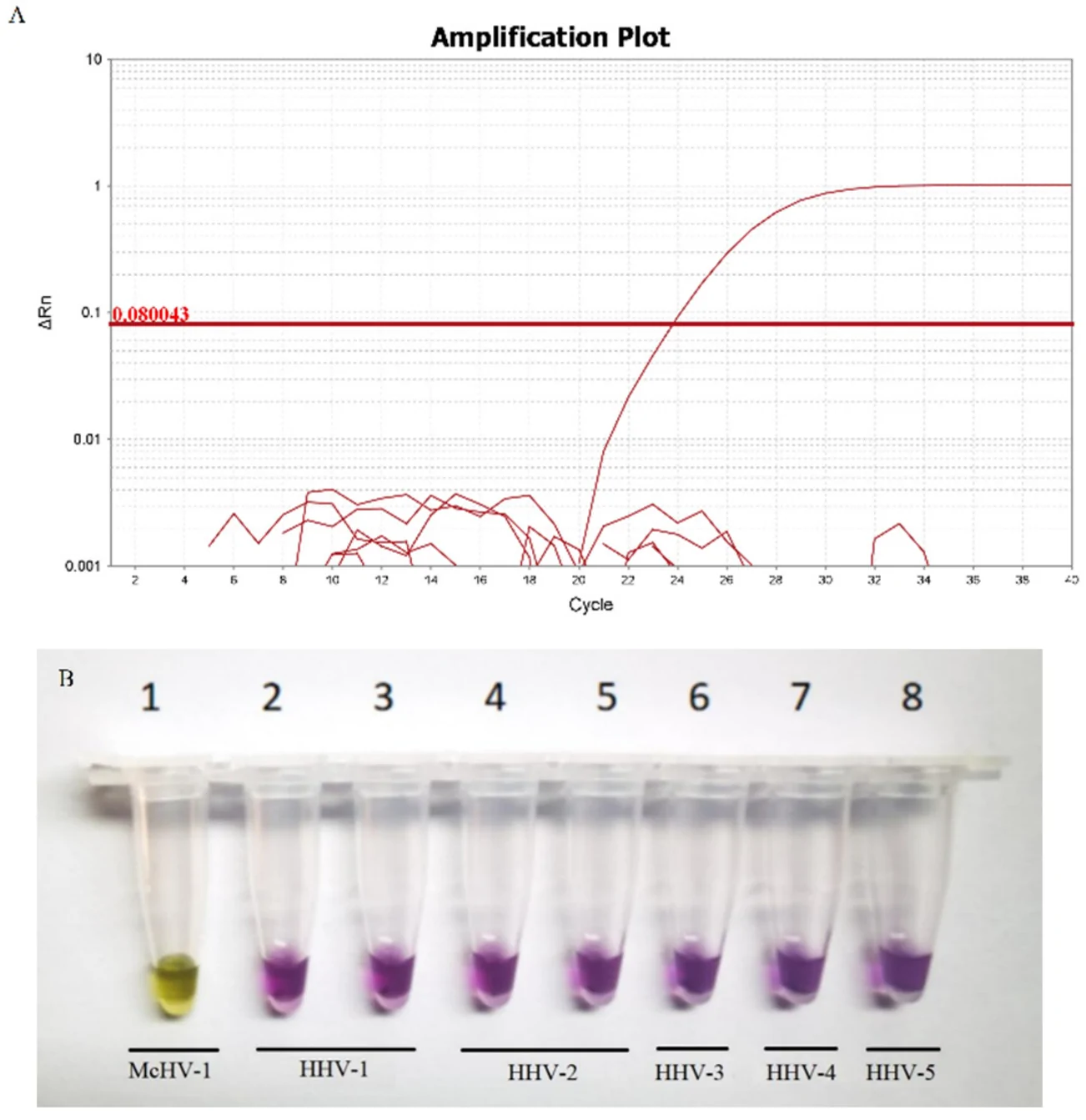
Analytical specificity of the 70 °C-LAMP assay against related herpesvirus strains. (**A**) Specificity was evaluated on a real-time PCR instrument using target McHV-1 alongside various ATCC reference strains of HHV-1 to HHV-5. The amplification plot demonstrates exclusive detection of the McHV-1 target (curve 1), which crossed the fluorescence threshold rapidly. Non-target reference viruses yielded no positive signals within the valid detection window. Note: The assay was performed under isothermal conditions (70 °C); the x-axis “Cycle” corresponds to the reaction time in minutes (1 cycle = 1 min). (**B**) Representative image of the colorimetric assay. The accumulation of pyrophosphate byproducts in the positive reaction (Tube 1) led to a visible color shift from deep violet to yellow-green, whereas negative samples (Tubes 2–8) remained violet. This visual distinction is consistent with the real-time fluorescence data. 1, McHV-1/BV; 2, HHV-1 (ATCC VR-1493); 3, HHV-1 (ATCC VR-733); 4, HHV-2 (ATCC VR-734); 5, HHV-2 (ATCC VR-1779); 6, HHV-3 (ATCC VR-916); 7, HHV-4 (ATCC VR-1492); 8, HHV-5 (ATCC VR-538).

To evaluate the analytical specificity of the 70 °C-LAMP assay, cross-reactivity was tested against a comprehensive panel of non-target human herpesviruses (HHV-1 through HHV-5). Positive real-time amplification signals were robustly and exclusively observed for the McHV-1 template. In contrast, all non-target viral strains and the no-template control (NTC) remained strictly below the fluorescence threshold within the ≤35-cycles diagnostic cutoff window, confirming high analytical specificity (Figure 4A). In the separate visual L-HNB colorimetric format (Figure 4B), accumulation of pyrophosphate byproducts in the positive McHV-1 reaction drove an unambiguous, temperature-stable color shift from deep violet to yellow-green. In contrast, all non-target HHV controls and NTC remained distinctly violet without any late false-positive color transition throughout the 30 min window, allowing for straightforward naked-eye discrimination.

### 3.3. Analytical Matrix-Tolerance Testing in Spiked Mock Samples

**Figure 5:**
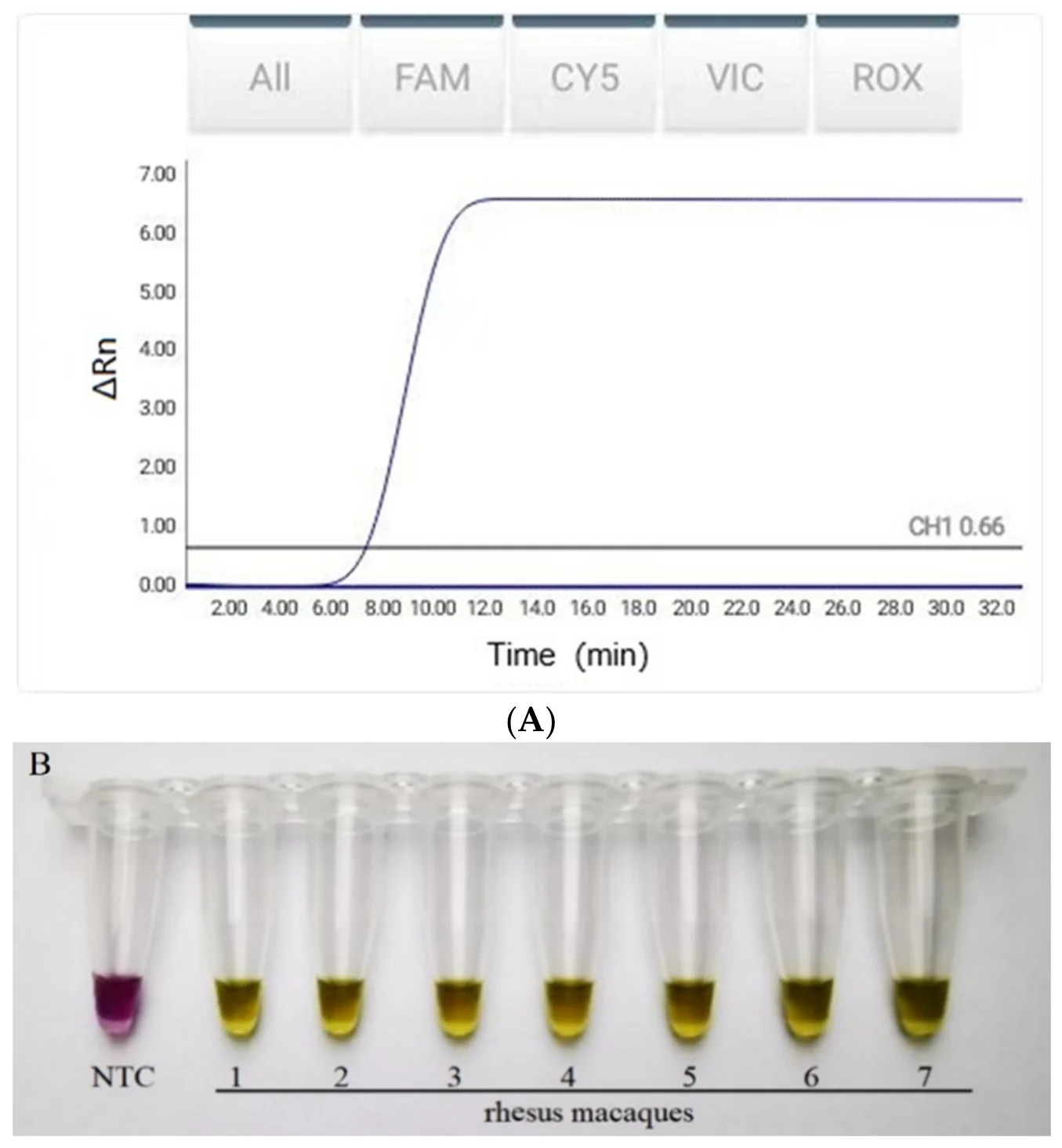
Analytical matrix tolerance and robustness of the HotStart LAMP assays in spiked mock saliva samples. (**A**) Real-time amplification kinetics performed on a portable isothermal amplification platform (YP-R16) demonstrating robust amplification of McHV-1 DNA (104 copies/μL) in unpurified macaque saliva within an established 30 min diagnostic cutoff. (**B**) End-point visual colorimetric detection in a 70 °C water bath using pre-added L-HNB dye. The tube on the far left represents the no-template control (NTC, remaining violet), and the subsequent seven tubes represent crude saliva specimens from seven individual macaques spiked with McHV-1 DNA, all yielding clear positive color transition to yellow-green. Matched unspiked saliva samples consistently remained violet (negative).

To evaluate the analytical matrix tolerance and resilience of the HotStart LAMP assay prototype against complex biological secretions, mock specimens were prepared by spiking McHV-1 viral DNA into unpurified macaque saliva. Real-time LAMP reactions were evaluated on a compact, portable isothermal platform (YP-R16) to test compatibility with field-deployable instrumentation (Figure 5A). Real-time amplification kinetics evaluated at a target spike concentration of 104 copies/μL revealed that McHV-1 targets consistently crossed the threshold within 10–18 min, remaining unaffected by the complex salivary milieu. These results confirm robust analytical matrix tolerance at the evaluated target concentration, while comprehensive evaluation across full dilution series in diverse clinical specimens will be pursued in future diagnostic studies.

Furthermore, to evaluate instrument-free visual capability in complex matrices, the L-HNB colorimetric assay was executed using a standard water bath maintained at 70 °C. The presence of unpurified salivary constituents across seven distinct macaque samples did not impede the clarity of the L-HNB colorimetric transition. All McHV-1-spiked saliva samples exhibited a distinct and unambiguous color change from deep violet to yellow-green within 20–30 min (Figure 5B). Crucially, matched unspiked saliva controls and NTC remained stably violet throughout the 30 min incubation, showing zero matrix-induced false color change. This clear visual readout demonstrates the assay’s robust tolerance against crude oral matrix inhibitors, supporting its potential utility as a simplified prototype for future frontline surveillance once validated on natural clinical cohorts.

## 4. Discussion

The development of an accessible molecular screening tool for McHV-1 is of paramount importance given its high case-fatality rate and recent zoonotic spillover events [3,13,14]. In this study, we successfully established a 70 °C HotStart LAMP assay that addresses the fundamental limitations of existing detection methodologies, delivering qPCR-level sensitivity (100 copies/reaction) and high specificity within 15 min.

The McHV-1 genome exhibits an exceptionally high GC content of approximately 74.5% to 75% [13,15]. This extreme GC bias presents a formidable hurdle for molecular diagnostics, particularly in isothermal amplification techniques such as LAMP [16]. In such complex multi-primer reaction systems, GC-rich targets frequently induce complicated secondary structures and unanticipated primer dimers, severely compromising amplification efficiency and driving frequent non-specific amplification or false-positive results [16,17]. To date, established molecular detection methods have predominantly targeted genes such as the highly conserved gB (*UL27*), gD (*US6*) [18], the major DNA-binding protein (*UL29*) [19], DNA polymerase (*UL30*), or the less conserved gG (*US4*) [14,16,17,20]. Unlike previous methodologies targeting these notoriously GC-dense open reading frames [13], our study introduces a novel strategy by selectively targeting the *UL1* glycoprotein gene. Although the *UL1* gene overall shares the high GC content characteristic of the McHV-1 genome, our comprehensive sequence alignment identified a highly conserved segment within this gene that possesses a localized, significantly more moderate GC content (61–62%). Crucially, this specific region is of sufficient length to facilitate the optimal design of both LAMP and qPCR primers. By deliberately circumventing the pan-genomic high-GC trap, our localized *UL1*-targeted approach effectively mitigates the propensity for primer dimerization and non-specific binding. Consequently, this strategic target selection substantially enhances the analytical specificity and overall robustness of the isothermal amplification assay.

Beyond target selection, a prominent shortcoming of conventional LAMP technology is its susceptibility to non-specific amplification, which may yield false-positive results driven by mispriming and primer dimerization during room-temperature reaction assembly [16,17]. To address this technical bottleneck, our methodology incorporates an aptamer-based HotStart Bst 3.2 DNA polymerase coupled with an elevated reaction temperature. According to manufacturer specifications, the aptamer sequesters and inhibits polymerase activity at ambient temperatures, which helps suppress premature primer interactions and the subsequent generation of primer dimers during reaction setup. Furthermore, rather than employing the traditional 60–65 °C isothermal range [16,17], our optimized assay is executed at an elevated temperature of 70 °C [21]. This elevated reaction temperature promotes aptamer dissociation to release polymerase activity while assisting in destabilizing the complex secondary structures inherent to the GC-rich McHV-1 target [16]. Accordingly, operating at 70 °C with HotStart chemistry may contribute to curtailing off-target amplification, which is consistent with the clean baselines and absence of non-specific amplification observed in our negative controls throughout the standardized 30 min analytical window.

Finally, the practical utility of this prototype is supported by the integration of closed-tube L-HNB visualization [22,23,24] and its demonstrated resilience in oral biological matrices. As shown by our mock sample testing, the colorimetric assay maintains stable visual distinction against background matrix interference from macaque mucosal secretions. This visual detection obviates the strict requirement for expensive thermal cyclers or optical detectors, highlighting its potential value as a simplified frontline tool for future McHV-1 surveillance in breeding colonies and post-exposure screening once validated on clinical cohorts [16,17]. 

A critical consideration in diagnostic design is the balance between assay inclusivity and analytical specificity. In this study, our *UL1*-targeted primers were strategically tailored to perfectly match McHV-1 strains originating from rhesus macaques, Japanese macaques, and bonnet macaques, which represent the vast majority of highly lethal zoonotic strains. Notably, we deliberately excluded strains derived from cynomolgus macaques. While a recent LAMP assay developed by Amano et al. sought broader pan-macaque coverage, the significant sequence divergence of cynomolgus-derived McHV-1 strains necessitates the use of complex multi-primer cocktails or degenerate sequences. In a 70 °C HotStart system designed for negligible background, introducing such primer complexity exponentially increases the thermodynamic risk of primer dimer formation, directly undermining field deployability. By prioritizing uncompromising specificity against the most high-risk strains, our assay ensures accurate diagnosis without false positives. Future iterations could employ a separate, parallel primer set tailored for cynomolgus strains, expanding host coverage without sacrificing the thermodynamic stability of the core assay.

Despite the robust performance of the 70 °C HotStart LAMP assay, we acknowledge three substantial limitations of the present study. First, the assay lacks an internal amplification control (IC) to monitor potential reaction inhibitors in crude matrix samples. Integration of an endogenous host reference gene (e.g., RNase P) into a multiplex format will be essential prior to field deployment. Second, testing relied on genomic DNA spiked into saliva matrix rather than live or inactivated viral particles, and thus evaluated matrix tolerance rather than viral lysis/extraction efficiency. This is partly due to the low natural viral shedding rate (~2%) and transient shedding window in asymptomatic macaque colonies. Third, analytical inclusivity is currently tailored for the McHV-1 core clade (rhesus and Japanese macaque isolates), and further evaluation across diverse macaque species variants is recommended.

In the management of specific-pathogen-free (SPF) macaque colonies, McHV-1 is mandated as one of the five essential screening pathogens. However, standard serological testing only indicates historical exposure, failing to distinguish between latent infection and active viral shedding. Therefore, direct nucleic acid detection is critical for identifying actively shedding individuals. While traditional PCR methodologies fulfill this need, their application is heavily constrained by the requirement for sophisticated thermal cycling equipment and highly trained personnel. This technical bottleneck is particularly problematic in actual macaque breeding facilities, where daily operations and animal handling frequently occur with minimal personal protective equipment (e.g., standard face masks). Thus, an accessible, low-barrier diagnostic tool like LAMP is urgently required to mitigate occupational exposure risks.

Although the current validation of our 70 °C HotStart LAMP assay relied primarily on spiked mock clinical specimens, this methodology was dictated by the pathogen’s exceptional biosafety constraints and unique pathobiology. As a highly lethal zoonotic agent, McHV-1 handling is strictly regulated: live virus culture requires BSL-4 containment, and in vivo infection models mandate Animal BSL-3 (ABSL-3) facilities. These stringent regulations severely limit the accessibility and cross-institutional transfer of naturally infected clinical materials. Furthermore, viral shedding in natural macaque hosts is notoriously intermittent and low-titer, making the acquisition of confirmed positive field samples logistically challenging.

To circumvent these hurdles while maintaining strict diagnostic rigor, we utilized unpurified, raw macaque saliva—which can be safely processed under standard BSL-2 conditions—as the biological background matrix. This approach arguably provides a more stringent evaluation of the assay’s field utility than using purified DNA, as it directly tests the system’s resilience against potent inhibitory components, such as nucleases and mucins, inherently present in crude oral secretions. The validation using spiked clinical matrices is a broadly accepted benchmark for point-of-care testing platforms targeting high-consequence, low-prevalence pathogens when natural specimens are inaccessible. Ultimately, our findings demonstrate that the 70 °C HotStart LAMP system remains highly sensitive and robust within a complex biological milieu, demonstrating strong potential as an analytical prototype for frontline McHV-1 surveillance and biosafety management in resource-limited settings. Although future studies incorporating naturally infected clinical samples will further validate its diagnostic accuracy, the robust performance of this HotStart LAMP assay supports its potential value as a robust analytical prototype for future McHV-1 surveillance and biosafety management upon diagnostic validation with clinical cohorts [16,17,25].

## 5. Conclusions

In conclusion, this study demonstrates a proof-of-concept analytical validation of a HotStart LAMP assay targeting the UL1 gene of McHV-1. The assay achieves high analytical sensitivity (preliminary practical analytical LOD of 100 copies/reaction in triplicate evaluation) and strict specificity without cross-reactivity against human herpesviruses or host macaque genomic DNA. As a single-target analytical prototype, further integration of an internal control and validation with clinical specimens will pave the way for its future field diagnostic application. However, as the present study was designed as an analytical matrix-tolerance validation using spiked specimens, its direct applicability to human infection diagnosis or field clinical screening remains to be established through future validation with naturally infected clinical specimens.

## Data Availability

The data that support the findings of this study are available from the corresponding author upon reasonable request.

## Abbreviations

The following abbreviations are used in this manuscript:

McHV-1	*Macacine alphaherpesvirus* 1
L-HNB	Leuco-Hydroxynaphthol Blue
LAMP	Loop-mediated Isothermal Amplification
LOD	Limit of detection
